# Prevalence and correlates of substance use among health care students in Nepal: a cross sectional study

**DOI:** 10.1186/s12889-017-4980-6

**Published:** 2017-12-12

**Authors:** Bimala Panthee, Suresh Panthee, Saroj Gyawali, Norito Kawakami

**Affiliations:** 1 Patan Academy of Health Sciences, Lalitpur Nursing Campus, Sanepa, Lalitpur Nepal; 2 Research and Development Centre, Kathmandu, Nepal; 3 Sustainable Study and Research Institute, Kathmandu, Nepal; 4 Graduate School of Biomedical Sciences, Tokyo Medical and Dental University, Tokyo, Japan; 5 Faculty of Environmental Management, Prince of Songkla University, Hat Yai, Thailand; 6 Department of Mental Health, Graduate School of Medicine, The University of Tokyo, Tokyo, Japan

**Keywords:** Health care students, Prevalence, Substance use

## Abstract

**Background:**

Substance use among health care students threatens professional standards and the delivery of quality services, potentially placing the public at risk. Therefore, our study aims to determine the prevalence and correlates associated with substance use among Nepalese health care students.

**Method:**

A cross-sectional survey using a self-administered health professional questionnaire was conducted among pharmacy, nursing, and public health students at three universities in Nepal in 2010.

**Results:**

We analyzed data from 407 respondents (response rate, 82%) with a mean age of 22 years (standard deviation = 3.71). The overall lifetime prevalence of substance use (i. e., illegal use of prescription drugs and illegal drug use) was 42.8%. Marijuana was the most commonly used illegal drug (8.8%) and minor opiates (e.g., codeine cough syrups) were the most widely used illegal prescription drugs (32.4%). Substance use was directly associated with cigarette smoking, peer influence, and heavy drinking. In addition, respondents reported some major and minor dysfunctions because of their substance use.

**Conclusion:**

The prevalence of substance use among health care students at the three universities in Nepal was high. Peer influence, cigarette smoking, and heavy drinking were significant predictors of substance use.

**Electronic supplementary material:**

The online version of this article (10.1186/s12889-017-4980-6) contains supplementary material, which is available to authorized users.

## Background

Substance use is defined as the continued use of substances, illegal drugs, or the misuse of prescription or over-the-counter drugs with negative consequences [1]. The lifestyle of health care professionals plays an important role and has a significant influence on the lifestyle of patients or clients [2]. No health care professional is immune to substance use (e.g., illegal use of prescription drugs and illegal drug use). Substance use by the health care professionals might influence their professional behavior and threaten the standard of the delivered health care services, potentially placing the public at risk [3]. Abuse, often begins during adolescence in college [4–6] and early beginners are at the higher risk of psychosocial problems, such as behavior patterns, psychiatric disorders, family system, peer relationships, leisure/recreation, and work adjustment [7]. Furthermore, this is associated with the failure to stop using substances on one’s own. Therefore, to promote the health of health care professionals and the quality of the future health care services provided, it is essential to assess health care students’ substance use.

Overall, the prevalence of substance use among health care students ranges from 5% to 67% [8–14], with higher percentages (67%) being reported in developed countries like the United States [8]. Among developing countries, such as Iran [9, 10], Ethiopia [11], Nepal [12, 13], and India [14] substance use among health care students varies from 5% to 48%. Moreover, 91.7% of the medical students using illicit substances admitted using them despite knowing their ill effects and the legal consequences [14]. In Nepal, substance use by medical students ranges from 12.8% to 15% for cannabis [12, 13], 4% for hashish, 1.12% for lysergic acid diethylamide, 0.22% for opioids, and 0.22% for amphetamine [13]. With a few exceptions [8, 15], most of the previous studies did not investigate multiple types of substance use among multiple groups of students [9, 11, 16]. For instance, Ahmadi et al. focused on opium use among medical students in southern Iran [9] and Khanal et al. studied only on medical students in Nepal, although different substances (i.e., alcohol, tobacco, cannabis) were included [13]. Similarly, Deressa and Azazh studied khat (*Catha edulis*) chewing among medical students in Ethiopia [11].

The use of marijuana and heroin together with the misuse of psychoactive and sedative drugs is of great national concern in Nepal [17]. In 1998, the annual prevalence of cannabis abuse was 3.2% [18]. In 2006, the estimated prevalence of opiate abuse was to be 0.3% in the Nepalese adult population aged 15–64 years [18]. A large survey conducted in 18 out of 75 districts by the Ministry of Home Affairs identified 91,534 hard drug users, with more than 83% being polydrug users [19]. Cannabis and opiates were the most commonly used drugs. In decreasing order, the user preference was for cannabis, heroin, pain medicine, and cough syrup [19]. The annual growth rate of drug users is 11.4%, and nearly three-fourths (73.1%) of the current drug users had their first drug intake before they were 20 years old [20]. In addition, there was a rise in the rate of injectable drug use between 2008 and 2011 [21], with 57% of the drug users being injectable drug users in 2013 [19]. This situation was further exacerbated by the fact that 13% of the injectable drug users shared needles with someone else.

Taken together, there is a strong need to assess substance use among health care students. Therefore, this study was conducted to determine the extent of prevalence of substance use among health care students. In addition, we aimed to examine the correlates of substance use among health care students in the Nepalese context. The results of this study are expected to contribute to design preventive programs, primarily focusing on the health care students.

## Methods

### Study design

In 2010, we carried out a cross-sectional survey of undergraduate pharmacy, nursing, and public health students at three universities (i.e., Tribhuvan University, Pokhara University and Purbanchal University) of Nepal.

### Study population and sample

All students taking pharmacy, nursing, and public health courses at those three universities were the study population. However, we purposively selected colleges and the students’ academic year for our data collection, which limited our target population to 555. Based on the students’ presence at the time of the survey, 499 students were asked to voluntarily participate in a lecture room setting and return the questionnaire in the same setting. Altogether, 498 responses were received (Additional file 1: Table S1). Excluding all the missing data, a total of 407 responses, i.e., 73.3% of target population, were used for the final analysis.

### Survey measures

Data were collected using a modified version of the Health Professional Questionnaire developed by Kenna and Wood [15]. The survey instrument was pilot tested for face validity with a group of ten volunteers from the first-year medical students of Tribhuvan University, Nepal. No major changes were required on the questionnaire. The four-page final version of the survey contained information about basic demographic characteristics and details of substance use including the effects of the substance use, if any.

### Demographic characteristics

We asked respondents about their age, gender, faculty, year and university of the study, active participation in religion, family history of alcohol, and family history of drug use.

### Cigarettes and alcohol use

We asked about cigarettes and alcohol use. Cigarette smoking and heavy drinking (5 or more drinks at one occasion) were asked as “how many times, if ever, have you smoked cigarettes?” and “how many times, if ever, have you had 5 or more drinks on the occasion?” respectively, for lifetime cigarette smoke and alcohol use. The rating was from “0” times to more than “30” times.

Participants were also asked about the number of times they received offers of alcoholic beverage and legal psychotherapeutic drugs (e.g., opiates, stimulants) by their friends and alcohol from pharmaceutical companies during the past year. They were also asked whether they had worked with a co-worker or a colleague who accepted self-medication with psychoactive drugs during the past year.

### Illegal drug use

Participants were asked about their lifetime experience and the tendency of use of each of three commonly found street drugs (i.e., marijuana, cocaine, and designer drugs). For instance, “how many times, if ever, have you smoked marijuana or hashish?” In Nepal, both marijuana and hashish are commonly found street drugs. Respondents were asked to rate the frequency of use, using response option “0” times to “>30 times” in their lifetime, the past year, and the past month. These three substances were included in a single group as “illegal drugs”. If the students used any of the illegal drugs, at least once in their lifetime, past year, and past month, they were regarded as illegal drug users during their lifetime, in the past year, and the past month, respectively. The lifetime, past year, and past month use of the illegal drugs was computed.

### Illegal use of prescription drug

A total of seven groups of prescription drugs (major opiates, minor opiates, stimulants, sedative-hypnotics, tranquilizers, anxiolytics and other prescription pain medicines) with examples were provided to assess drug use without authorization or prescription. We asked, “How many times, if ever, have you used following medications/drugs on your own authorization or for uses other than the intended use?”. To facilitate identification, examples of generic and brand names were provided for each medication class. Response options and duration of use were the same as that for illegal drug use. Similar to the operational definition of illegal drug users, if the students used any of the prescription drugs at least once in their lifetime, in the past year, and in the past month they were regarded as illegal users of the prescription drug during their lifetime, the past year, and the past month, respectively.

We further asked whether the use of illegal drugs or prescription drugs without a prescription caused any dysfunction in their daily activities or life using eight questions. We further subdivided these eight questions into “minor dysfunction” (initial four questions) and “major dysfunction” (following four questions) [15]. The list of dysfunctions caused by the drug use were as follows: (1) falling behind in work; (2) calling in sick or being late; (3) having trouble getting along with people; (4) worrying that you might be using too much or too often; (5) seriously considered suicide; (6) having an auto accident or other type of accident; (7) providing less than your best patient care performance; and (8) seeing a psychiatrist, psychologist, or a counselor. The example of minor dysfunction was “has your drug use ever caused you to get behind in your work?” Similarly, an example of major dysfunction was “has your drug use ever caused you to seriously consider suicide?”

All potential respondents were provided with the participant’s information sheet about the study. Students were asked to complete the survey instrument in a ecture room setting voluntarily and anonymously and return the questionnaire in the same setting. No incentive was provided for completing the questionnaire. The study was approved by the Ethical Review Board of the Institute of Medicine, Tribhuvan University, Kathmandu, Nepal.

### Statistical analysis

All data were analyzed using SPSS software (version 20; Armonk, NY, USA). Cases with any missing data were excluded from the analysis. Demographic variables and substance use were analyzed using descriptive statistics. The frequency of substance use was recorded as 0 = never used, and 1 = any use (i.e., 1 time to >30 times use). Adjusted logistic regression was applied to identify the risk factors for substance use. Chi-square test was used to examine any differences in substance use by demographic characteristics and faculty (pharmacy, nursing and public health). Fisher’s exact test was applied for cell count less than 5. *P* value for statistical significance was set as less than 0.05.

## Results

### Sample

Completed responses were received from 498 respondents. However, only 407 responses were used for the final analysis because of the missing data, with a response rate of 73.3%. Respondents were from Tribhuvan University (50.4%), Pokhara University (28.7%) and Purbanchal University (20.9%), with the mean age of 22.2 years (standard deviation [SD] = 3.7). Among them, 37.8% majored in pharmacy with a mean age of 21.1 years (SD = 1.9), 39.1% majored in nursing with a mean age of 23.7 years (SD = 4.9) and 23.1% majored in public health with a mean age of 21.4 years (SD = 2.4). Nursing students were all women. Detailed demographic characteristics are presented in Table 1.

**Table 1:** Demographic characteristics and previous encounters with cigarette, alcohol, and drugs (*N* = 407)

Demographic variables	Total (N = 407) n (%)	Pharmacy students (*n* = 154) n (%)	Nursing students (*n* = 159) n (%)	Public health students (*n* = 94) n (%)	*P* value ^a^
Age group (years)					
16–20	143 (35.1)	67 (43.5)	37 (23.3)	39 (41.5)	<.01
21–25	215 (52.8)	83 (53.9)	83 (52.2)	49 (52.1)	
> 25	49 (12.0)	4 (2.6)	39 (24.5)	6 (6.4)	
Sex					
Male	154 (37.8)	104 (67.5)	–	50 (53.2)	<.01
Female	253 (62.2)	50 (32.5)	159 (100)	44 (46.8)	
Active participation in religion					
Yes	255 (62.7)	86 (55.8)	107 (67.3)	62 (66.0)	.08
No	152 (37.3)	68 (44.2)	52 (32.7)	32 (34.0)	
Family history of alcohol abuse problem					
Yes	84 (20.6)	27 (17.5)	37 (23.3)	20 (21.3)	.44
No	323 (79.4	1247(82.5)	122(76.7)	74 (78.7)	
Family history of drug abuse problem					
Yes	24 (5.9)	9 (5.8)	6 (3.8)	9 (9.6)	.16
No	383 (94.1)	145 (94.2)	153 (96.2)	85 (90.4)	
Offer of alcoholic beverage by friends					
Yes	187 (45.9)	85 (55.2)	46 (28.9)	56 (59.6)	<.01
No	220 (54.1)	69 (44.8)	113 (71.1)	38 (40.4)	
Offer of alcohol by pharmaceutical company					
Yes	62 (15.2)	29 (18.8)	24 (15.1)	9 (9.6)	.14
No	345 (84.8)	125 (81.2)	135 (84.9)	85 (90.4)	
Offer of drugs by friends					
Yes	24 (5.9)	12 (7.8)	5 (3.1)	7 (7.4)	.16
No	383 (94.1	142 (92.2)	154 (96.9)	87 (92.6)	
Experience of working with coworker or colleague who accepted themselves as self- medication					
Yes	43 (10.6)	23 (14.9)	9 (5.7)	11 (11.7)	.02
No	364 (89.4)	131 (85.1)	150 (94.3)	83 (88.3)	
Drinking 5 or more at one sitting in one occasion					
Yes	106 (26)	52 (33.8)	28 (17.6)	26 (27.7)	<.01
No	301 (74)	102 (66.2)	131 (82.4)	68 (72.3)	
Cigarette smoking during lifetime					
Yes	88 (21.6)	47 (30.5)	20 (12.6)	21 (22.3)	<.01
No	319 (78.4)	107 (69.5)	139 (87.4)	73 (77.7)	

^a^Based on Chi-square test among three groups (pharmacy, nursing and public health students)

### Prevalence of substance use

The overall prevalence of substance use (illegal use of prescription drugs and illegal drug use) was 42.8% during lifetime; 26% in the past year, and 11.3% in the past month.

### Reported illegal drug use

Overall, 8.8% of respondents used illegal drugs (i.e., marijuana, cocaine, and designer drugs) during their lifetime (Table 2); prevalence was 5.2% in the past year and 3.2% in the past month. Higher lifetime prevalence was observed among pharmacy students and public health students than nursing students; the difference being statistically significant (χ^2^ = 16.15, *p* < 0.01). Similarly, men (19.5%) reported higher lifetime illegal drug use than women did (2.4%) (χ^2^ = 34.75, p < 0.01).

**Table 2:** Prevalence of substance use during lifetime and its demographic correlates^a^

Demographic variables	Illegal drug use n (%)	Illegal use of prescription drug n (%)
Total (N = 407)	36 (8.8)	157 (38.6)
Age group (years)		
16–20 (*n* = 143)	12 (8.4)	56 (39.2)
21–25 (*n* = 215)	18 (8.4)	84 (39.1)
> 25 (*n* = 49)	6 (12.2)	17 (34.7)
	*P* = 0.67	*P* = 0.83
Sex		
Female (*n* = 253)	6 (2.4)	105 (41.5)
Male(n = 154)	30 (19.5)	52 (33.8)
	P < 0.01 (χ^2^ = 34.75)	*P* = 0.08
Faculty		
Pharmacy (n = 154)	22 (14.3)	53 (34.4)
Nursing (n = 159)	3 (1.9)	73 (45.9)
Public Health (n = 94)	11 (11.7)	31 (33.0)
	P < 0.01 (χ^2^ = 16.15)	*P* = 0.05 (χ^2^ = 5.97)
Active participation in religion		
Yes (*n* = 255)	15 (5.9)	93 (36.5)
No (*n* = 152)	21 (13.8)	64 (42.1)
	P < 0.01 (χ^2^ = 7.43)	*P* = 0.23
Family history of alcohol abuse problem		
Yes (*n* = 84)	9 (10.7)	32 (38.1)
No (*n* = 323)	27 (8.4)	125 (38.7)
	*P* = 0.49	*P* = 0.76
Family history of drug abuse problem		
Yes (*n* = 24)	4 (16.7)	6 (25.0)
No (*n* = 383)	32 (8.4)	151 (39.4)
	*P* = 0.16	*P* = 0.14
Offer of alcohol by friends		
Yes (*n* = 187)	33 (17.6)	76 (40.6)
No (*n* = 220)	3 (1.4)	81 (36.8)
	P < 0.01 (χ^2^ = 33.24)	*P* = 0.54
Offer of alcohol by pharmaceutical company		
Yes (*n* = 62)	10 (16.1)	27 (43.5)
No (*n* = 345)	26 (7.5)	130 (37.7)
	*P* = 0.02 (χ^2^ = 4.81)	*P* = 0.28
Offer of drugs by friends		
Yes (n = 24)	7 (29.2)	12 (50.0)
No (n = 383)	29 (7.6)	145 (37.9)
	P < 0.01 (χ^2^ = 13.06)	*P* = 0.25
Experience of working with coworker or colleague who accepted self-medication with psychoactive drug		
Yes (*n* = 43)	9 (20.9)	21 (48.8)
No (*n* = 364)	27 (7.4)	136 (37.4)
	P < 0.01 (χ^2^ = 8.70)	P = 0.16
Five or more drink at one time in one occasion during lifetime		
Yes (*n* = 106)	26 (24.5)	53 (50.0)
No (*n* = 301)	10 (3.3)	104 (34.6)
	P < 0.01 (χ^2^ = 43.72)	*P* < 0.01 (χ^2^ = 7.89)
Cigarette smoking during lifetime		
Yes (*n* = 88)	29 (33.0)	44 (50.0)
No (*n* = 319)	7 (2.2)	113 (35.4)
	P < 0.01 (χ^2^ = 80.94)	*P* = 0.01 (χ^2^ = 6.18)

aChi-square test was used to show differences in the prevalence among groups classified based on demographic variables. Fisher’s exact test was used if any cells had an expected count less than 5

Marijuana was the most commonly used illegal drug for pharmacy and public health students (13% and 10.6%, respectively), while only 1.3% (2/159) of nursing students reported using marijuana during their lifetime (χ^2^ = 16.15, p < 0.01) (Table 3). Pharmacy students used a significantly higher amount of designer drugs (χ^2^ = 10.50, *p* = 0.005) than did public health students, while none of the nursing students reported using them during their lifetime.

**Table 3:** Prevalence of substance use during lifetime, in the past year and in the past month among Nepalese healthcare students (N = 407)

Substances	Lifetime n (%)	Past year n (%)	Past month n (%)	Pharmacy students n (%) N = 154			Nursing students n (%) N = 159			Public Health students n (%) N = 94		
				Life time	Past year	Past month	Lifetime	Past year	Past month	Life time	Past year	Past month
Marijuana	32 (7.9)	18 (4.4)	10 (2.5)	20** **(13)**	14** **(9.1)**	8* **(5.2)**	2 (1.3)	0 (0)	1 (.6)	10 (10.6)	4 (4.4)	1 (1.1)
Cocaine	4 (1)	3 (.7)	3 (.7)	3 (1.9)	2 (1.3)	2 (1.3)	1 (0.6)	1 (0.6)	0 (0)	0 (0)	0 (0)	1 (1.1)
Designer drug	9 (2.2)	3 (.7)	2 (.5)	8** **(5.2)**	2 (1.3)	1 (0.6)	0 (0)	0 (0)	0 (0)	1 (1.1)	1 (1.1)	1 (1.1)
Minor opiates	132 (32.4)	68 (16.7)	26 (6.4)	43 (27.9)	23 (14.9)	9 (5.8)	62 (39.0)	27 (17.0)	11 (6.9)	27 (28.7)	18 (19.1)	26 (6.4)
Major opiates	7 (1.7)	2 (.5)	5 (1.2)	3 (1.9)	2 (1.3)	3 (1.9)	4 (2.5)	0 (0)	2 (1.3)	0 (0)	2 (0.5)	0 (0)
Stimulants	4 (1)	4 (1)	4 (1.0)	2 (1.3)	3 (1.9)	3 (1.9)	0 (0)	0 (0)	0 (0)	2 (2.1)	1 (1.1)	1 (1.1)
Sedatives	10 (2.5)	7 (1.7)	6 (1.5)	7* **(4.5)**	4 (2.6)	4 (2.6)	0 (0)	1 (0.6)	0 (0)	3 (3.2)	2 (2.1)	2 (2.1)
Tranquilizer	9 (2.2)	7 (1.7)	4 (1.0)	4 (2.6)	3 (1.9)	3 (1.9)	2 (1.3)	2 (1.3)	0 (0)	3 (3.2)	2 (2.1)	1 (1.1)
Anxiolytics	32 (7.9)	17 (4.2)	5 (1.2)	12 (7.8)	6 (3.9)	3 (1.9)	12 (7.5)	8 (5.0)	1 (0.6)	8 (8.5)	3 (3.2)	1 (1.1)
Pain medicine	63 (15.5)	31 (7.6)	10 (2.5)	23 (14.9)	7** **(4.5)**	2 (1.3)	31 (19.5)	22 (13.8)	6 (3.8)	9 (9.6)	2 (2.1)	2 (2.1)

Chi-square test for group differences *p < .05, **p < .01, significant results are presented in bold fonts

### Reported illegal use of prescription drugs

Overall, 38.6% of respondents reported the use of any kind of prescription drug without prescription in their lifetime followed by 22.6% in the past year and 9.6% in the past month. A higher percentage of nursing students (45.9%) reported the use of illegal prescription drug (in total) during their lifetime compared with pharmacy (34.4%) and public health students (33.0%). There was no significant gender difference in the frequency of illegal use of prescription drugs (Table 2).

Minor opiates (e.g., codeine cough syrups) was the most commonly misused prescribed drug (27.9%, 39.0%, and 28.7%) by pharmacy, nursing and public health students, respectively followed by pain medicine (e.g., Temazepam, Flurazepam) (Table 3). The difference among groups was not statistically significant. While all groups of health care students reported using a variety of prescribed drugs, a significant between-group difference was observed only for the use of sedatives during lifetime (χ^2^ = 7.01, *p* = 0.03); with pharmacy students being more likely to use sedatives. The rate of lifetime prescription drug use by pharmacy students ranged from 1.3% for stimulants to 27.9% for minor opiates. None of the nursing students reported the use of stimulants and sedatives during their lifetime. Similarly, none of the pharmacy students reported the use of major opiates during their lifetime.

In addition, 41 (10.1%) of the respondents reported that they suffered from one or all of the four minor dysfunctions because of their use of drugs. Similarly, 15 (3.7%) students reported major dysfunctions because of their drug use. A higher number of pharmacy students reported both major (5.8%) and minor dysfunctions (18.8%) as a result of drug use; however, a significant difference was observed only for minor dysfunction.

### Correlates

After adjusting for the effect of demographic variables, we found that cigarette smoking during lifetime was the most significant variable predicting illegal drug use (odds ratio [OR] = 10.33, confidence interval [CI] = 3.46, 30.79). Similarly, offer of drugs by friends (OR = 5.77, CI = 1.25, 26.49), offer of alcoholic beverages by friends (OR = 4.28, CI = 1.01, 18.02), and heavy drinking during lifetime (OR = 2.69, CI = 3.46, 30.79) also predicted illegal drug use. In addition, cigarette smoking and heavy drinking were the significant predictors of illegal use of prescription drugs (Table 4).

**Table 4:** Factors affecting lifetime illegal drug use and illegal use of prescription drugs

Independent variables	Illegal drug use		Illegal use of prescription drug	
	Odds	CI	Odds	CI
Age group (years)				
16–20	0.36*	0.05, 2.19	1.87	0.87, 4.02
21–25	0.17	0.03, 0.86	1.43	0.71, 2.88
> 25	1.00		1.00	
Gender				
Male	2.77	0.49,15.47	0.67	0.35, 1.29
Female	1.00		1.00	
Faculty				
Pharmacy	0.85	0.27, 2.60	0.94	0.52, 1.67
Nursing	0.70	0.09, 5.27	1.91	0.99, 3.72
Public Health	1.00		1.00	
Active participation in religion				
Yes	0.94	0.35, 2.47	1.23	0.79, 1.93
No	1.00		1.00	
Alcohol problem in family				
Yes	0.79	0.23, 2.70	0.97	0.56, 1.67
No	1.00		1.00	
Drug problem in family				
Yes	0.30	0.04, 1.92	1.94	0.68, 5.49
No	1.00		1.00	
Offer of alcoholic beverage by friends				
Yes	4.28*	1.01, 18.02	1.005	0.60, 1.67
No	1.00		1.00	
Offer of any psychoactive drugs by friends				
Yes	5.77*	1.25, 26.49	1.21	0.48, 3.04
No	1.00		1.00	
Offer of alcohol by pharmaceutical company				
Yes	1.22	0.40, 3.75	1.21	0.66, 2.22
No	1.00		1.00	
Experience of working with coworker or colleague who accepted self-medication with psychoactive drug				
Yes	1.49	0.43, 5.19	1.56	0.77, 3.14
No	1.00		1.00	
Heavy drinking during lifetime				
Yes	2.69*	1.04, 6.95	1.81*	1.08, 3.03
No	1.00		1.00	
Cigarette smoking during lifetime				
Yes	10.33**	3.46, 30.79	1.87*	1.04, 3.36
No	1.00		1.00	

*p < .05, **p < .01, Logistic regression adjusted for age, gender, faculty, and participation in religion, family history of alcohol and drug problems. Variables were entered simultaneously in the model and significant results are denoted in bold fonts

## Discussion

To our knowledge, this is the first survey conducted in Nepal that included a broad range of substances and multiple groups of health care students. The overall prevalence of substance use among health care students was found to be 42.8% during their lifetime, even excluding alcohol and cigarette smoking. Peer influence, heavy alcohol drinking, and cigarette smoking were the significant predictors of substance use among Nepalese health care students.

Marijuana was the most commonly used illegal drug. The rate of use of marijuana in our study (7.9%) differed from an earlier study (12.8% - 15%) among Nepalese medical students [12, 13]. Pharmacy and public health students had a higher prevalence of illegal drug use during their lifetime. Pharmacy students used marijuana and designer drugs more frequently. However, only a few and no nursing students reported the use of marijuana and designer drugs, respectively, in their lifetime. This difference was less apparent after adjusting for demographic variables. Our results differ from a report of lower prevalence of marijuana and cocaine use observed for pharmacy students than nursing students in the US [5, 8]. The observed higher prevalence of illegal drugs among pharmacy and public health students in our study may be attributable to differential demographic characteristics, such as gender, because nursing students in our study were all women and none of them reported the use of designer drugs.

The most commonly used illegal prescription drugs were minor opiates (codeine cough syrups) (32.4%) followed by pain medicine (Tramadol, Pentazocine) (15.5%) in contrast to the other reports [9], where none of the Nepalese medical students used opioids. In the US, misuse of prescription-type drugs (e.g., amphetamine, opiate, sedative/hypnotic, tranquilizer, or inhalant) by nurses was 6.9% [22]. In our study, nursing students had a higher lifetime prevalence of illegal prescription drugs use than other groups. This pattern still remained after adjusting for demographic variables. Nursing students used minor opiates (e.g., codeine cough syrups) and pain medicine (e.g., tramadol, Pentazocine) more frequently than did pharmacy and public health students, although the difference was not statistically significant. These results seem consistent with the higher prevalence of opiates use observed for nursing students than pharmacy students in the US [8]. However, care should be taken with this comparison because our study population comprised female nursing students. In Iran, the most commonly used substance by nursing students was opium [10]. In contrast, pharmacy students were significantly more likely to report the use of sedatives or hypnotics during their lifetime than nursing and public health students. This finding is consistent with the use of benzodiazepam (3.5%) by male medical students in Nepal, whereas no female medical students used it [13]. However, this observation is inconsistent with the earlier US report [8]. Pharmacy students in our study were probably more stressed about their studies than nursing and public health students, which led them to use these drugs more often.

Students reported both major and minor dysfunctions because of their use of drugs. These dysfunctions were higher among pharmacy students. A higher proportion of major dysfunction among pharmacy students is similar to the higher proportion of major dysfunction among pharmacists [15], which suggests potential vulnerability in the health of future health care professionals and the quality of the health care services they provid. Thus, a proper actions should be taken to address this issue.

Even after adjusting for demographic variables, we found heavy drinking and cigarette smoking were found to be significant predictors for substance use, which suggests that preventive programs to reduce drinking and smoking habits in health care students should be launched to reduce the risk of further substance abuse. It should be taken into consideration that all those controlled variables were significant predictors of substance use in a previous study [8].

Concerning the association between peer influence and substance use, we found that peer influence was also a significant predictor. This is consistent with a study in Ethiopia [11], where peer influence was a significant predictor of substance use among medical students, and a national survey among drug users in Nepal, where 83% of drug users reported the reasons of drug use as peer pressure [20]. This finding suggested that peer support programs could be implemented to reduce substance use and promote the health care service they provide. In itself, however, this is a challenging task because the students themselves displayed a lack of awareness and suboptimal attitudes towards substance use [23].

The considerably high prevalence of substance use among health care students suggests the vulnerability of health care students. In addition, some of the respondents who reported substance use at some point in their lives, also attributed some amount of dysfunction in their daily lives to substance use. Therefore, our findings indicate a need to educate health care students about substance use in any form. They need to know: (1) that self-treatment with prescription drugs, no matter how minor or infrequent, is inappropriate and hazardous; (2) that accepting offer of alcoholic beverage and drugs by friends may predispose them to substance abuse; (3) that alcohol and smoking may be an indicator of substance use; and (4) the warning signs of psychological and physical addiction.

### Limitations

About 20% of the data were excluded from the final analysis because of the missing values. In Nepal, only women can study nursing; therefore, comparison of our results with other reports including both male and female students as nursing students would not be valid. In addition, we did not include the medical students in this study; therefore, the study findings cannot be generalized to all health care students. As this was a cross-sectional survey, no causal relationship could be inferred. We recommend a longitudinal study including the questions regarding dependency on substance and initiation of each substance use that we lacked in this study.

However, our study has some methodological strengths. It was a large survey including three major universities in Nepal with a range of health care students. The survey also included a broad range of substances. In addition, a high response rate (73% after eliminating the missing data) strengthens the generalizability of the study findings within these student groups.

## Conclusion

The prevalence of substance use in health care students (pharmacy, nursing and public health) is evident. Peer influence (e.g., offer of alcohol and drugs by friends), cigarette smoking, and heavy drinking are significant predictors of substance use among Nepalese health care students. Therefore, health care policy should address prevention of substance use through peer support programs among health care students.

## Additional file

Additional file 1:
**Table S1.** Total number of students and responses received. (DOCX 20 kb)
Additional file 2: Health Professional Questionnaire. (PDF 182 kb)
