# Supplementary material for: Prevalence and correlates of substance use among health care students in Nepal: a cross sectional study
Source: BMC Public Health. 2017 Dec 12;17:950. doi: 10.1186/s12889-017-4980-6 (PMC5727951; doi:10.1186/s12889-017-4980-6)
Supplement: Additional file 1: — Table S1. Total number of students and responses received. (DOCX 20 kb) [file 12889_2017_4980_MOESM1_ESM.docx]

**Supplementary Table 1. Total number of students and responses received**

| University | Program | Year | Total Students | Students present that day | Distributed questionnaires | Responses received | Response rate |
| --- | --- | --- | --- | --- | --- | --- | --- |
| Tribhuvan | BPharm | 1 | 20 | 18 | 18 | 18 |  |
|  | BPharm | 2 | 15 | 14 | 14 | 14 |  |
|  | BPharm | 3 | 16 | 15 | 15 | 15 |  |
|  | BPharm | 4 | 6 | 6 | 6 | 6 |  |
|  |  |  | **57** | **53** | **53** | **53** | **93%** |
|  | BN | 1 | 48 | 39 | 39 | 39 |  |
|  | BN | 2 | 48 | 46 | 46 | 46 |  |
|  |  |  | **96** | **85** | **85** | **85** | **88.6%** |
|  | BScNsg | 1 | 20 | 19 | 19 | 19 |  |
|  | BScNsg | 2 | 20 | 20 | 20 | 20 |  |
|  | BScNsg | 3 | 18 | 17 | 17 | 17 |  |
|  | BScNsg | 4 | 20 | 20 | 20 | 20 |  |
|  |  |  | **78** | **76** | **76** | **76** | **97.5%** |
|  | BPH | 1 | 20 | 14 | 14 | 14 |  |
|  | BPH | 2 | 20 | 17 | 17 | 17 |  |
|  | BPH | 3 | 20 | 16 | 16 | 16 |  |
|  |  |  | **60** | **47** | **47** | **47** | **78.3%** |
| Pokhara | BPharm | 1 | 40 | 38 | 38 | 38 |  |
|  | BPharm | 2 | 40 | 38 | 38 | 38 |  |
|  | BPharm | 3 | 38 | 35 | 35 | 35 |  |
|  | BPharm | 4 | 32 | 30 | 29 | 29 |  |
|  |  |  | **150** | **141** | **140** | **140** | **93.4%** |
| Purbanchal | BPH | 1 | 37 | 29 | 29 | 29 |  |
|  | BPH | 2 | 37 | 31 | 31 | 31 |  |
|  | BPH | 3 | .. | .. | .. | .. |  |
|  |  |  | **74** | **60** | **60** | **60** | **81.1%** |
|  | BScNsg | 1 | 20 | 18 | 18 | 18 |  |
|  | BScNsg | 2 | .. | .. | .. | .. |  |
|  | BScNsg | 3 | .. | .. | .. | .. |  |
|  | BScNsg | 4 | 20 | 19 | 19 | 19 |  |
|  |  |  | **40** | **37** | **37** | **37** | **92.5%** |

| Total Students | Distributed questionnaires | Total responses | Response rate (%) |
| --- | --- | --- | --- |
| 555 | 499 | 498 | 89.73 |

Nursing Students: Includes Post Basic Nursing (BN) and Bachelor of Science in Nursing (BScNsg)

Pharmacy Students; Bachelor of Pharmacy (BPharm)

Public Health Students: Bachelor of Publc Health (BPH)
