# Supplementary material for: Prevalence and correlates of substance use among health care students in Nepal: a cross sectional study
Source: BMC Public Health. 2017 Dec 12;17:950. doi: 10.1186/s12889-017-4980-6 (PMC5727951; doi:10.1186/s12889-017-4980-6)
Supplement: Additional file 2: — Health Professional Questionnaire. (PDF 182 kb) [file 12889_2017_4980_MOESM2_ESM.pdf]

Please answer the following questions by tick mark (√) or a cross (X) with a dark pen. Record your best guess if no answer fits the question exactly. We realize that certain portions of this survey ask questions of a sensitive nature. You do not need to fear about this as the result of this survey will be anonymous and confidential. Your honest answers are requested. Please don't put your name in the questionnaire.

Section: A

1. **Age (years): ………..**
2. **Gender:** ☐ 1. Male ☐ 2. Female
3. **University of study:…………………………………………….** **Faculty:………………………………….**
4. **Course you are currently undertaking:** ☐ 1. Bachelor ☐ 3. PhD ☐ 2. Masters ☐ 4. Other (please specify)…………..……..
5. **Year of study:** ☐ 1. First ☐ 2. Second ☐ 3. Third ☐ 4. Fourth ☐ 5. Others:…………………

6. **Do you actively participate in religion? (eg praying, meditation, spirituality, worship)** ☐ 1. Yes ☐ 2. No
7. **Which of the following best describes your practice of prayer or religious meditation? (answer only one):** ☐ 1. No influence ☐ 2. A small amount ☐ 3. Some ☐ 4. A fair amount ☐ 5. Strong influence
8. **Which of the following statements comes closest to your belief about God? (answer only one):**
   - ☐ 1. I am sure God really exists and is active in my life
   - ☐ 2. Although I sometimes question God's existence, I believe in God and believe he knows me as a person
   - ☐ 3. I don't know if there is personal God, but I believe in a higher power of some kind
   - ☐ 4. I don't know if there is personal God or a higher power of some kind, and I don't know if I will ever know
   - ☐ 5. I don't believe in personal God or in higher power
9. **Religion gives me a great amount of comfort and security in life:** ☐ 1. I strongly disagree ☐ 2. I disagree ☐ 3. I am uncertain ☐ 4. I agree ☐ 5. I strongly agree

10. **Do you think any of your family members has/had an alcohol abuse problem?** ☐ 1. Yes ☐ 2. No
11. **Do you think any of your family members has/had a drug abuse problem?** ☐ 1. Yes ☐ 2. No

**12. Please consider the number of times the following may have occurred to you during the past year:**	**Never**	**Seldom 1 or 2 times**	**Occasionally 3 to 5 times**	**Sometimes 6 to 9 times**	**Often 10 or more times**
a. How many times in the past year has a friend or colleague given, bought or offered you and alcoholic beverage?	☐	☐	☐	☐	☐
b. How many times in the past year has alcohol been given, bought or offered to you at seminars or dinners sponsored by pharmaceutical companies?	☐	☐	☐	☐	☐
c. How many times during the past year has a friend given you or offered you a legal psychotherapeutic drug (eg opiates, stimulants)	☐	☐	☐	☐	☐
d. How often during the past year have you worked with a co-worker or colleague who condoned or accepted self medication with psychoactive drugs?	☐	☐	☐	☐	☐

13.

a. I consider myself a:

- ☐ 1. Non drinker
- ☐ 2. Infrequent drinker
- ☐ 3. Light drinker
- ☐ 4. Moderate drinker
- ☐ 5. Heavy drinker
- ☐ 6. Problem drinker

b. During past year, in a typical week, on how many days did you have AT LEAST 1 DRINK containing ALCOHOL?

- ☐ 0 Days
- ☐ 1 Day
- ☐ 2 Days
- ☐ 3 Days
- ☐ 4 Days
- ☐ 5 Days
- ☐ 6 Days
- ☐ 7 Days

c. Please indicate the average number of drinks in one sitting during the past year

- ☐ 0
- ☐ 1
- ☐ 2
- ☐ 3
- ☐ 4
- ☐ 5
- ☐ 6
- ☐ 7
- ☐ 8

Section: B

1. Please indicate when, if ever, you first used the following substances without a prescription or for use other than intended. (See examples for each medication/drug class for reference)

	Never Tried	Before College	During College
a. Alcohol (includes all forms)	☐	☐	☐
b. Cigarettes (includes all tobacco products i.e. cigar, pipes etc)	☐	☐	☐
c. Marijuana (includes Hashish, THC, etc)	☐	☐	☐
d. Cocaine (includes all forms, manufactured, and all street acquired forms of cocaine, etc)	☐	☐	☐
e. Designer drugs (includes Ecstasy, GHB etc.)	☐	☐	☐
f. Hallucinogens (includes LSD, PCP etc)	☐	☐	☐
g. Major opiates (includes Fentanyl, Morphine etc)	☐	☐	☐
h. Minor opiates (includes Paracetamol with codeine, Paracetamol with Hydrocodone, Codeine cough syrups etc)	☐	☐	☐
i. Stimulants (includes Methamphetamine)	☐	☐	☐
j. Non narcotics Analgesics	☐	☐	☐
k. Anxiolytics (includes Diazepam, Lorazepam, Alprazolam etc)	☐	☐	☐
l. Sedative Hypnotics (includes Temazepam, Flurazepam, Zolpidem etc)	☐	☐	☐
m. Inhalants (includes Nitrous Oxide, Amyl Nitrate, Butyl Nitrate, Halothane etc)	☐	☐	☐
n. Antibiotics (includes Amoxycillin, Metronidazole etc.)	☐	☐	☐
o. Antidepressants (includes Fluoxetine, Amoxapine, Imipramine etc)	☐	☐	☐
p. Tranquilizers (includes Phenobarbital, Ketamine etc)	☐	☐	☐
q. Other prescription pain medicines (includes Tramadol, Butorphanol Pentazocine etc)	☐	☐	☐

2.
a. How many times, if ever, have you had 5 or more drinks on the occasion?

TIMES	0	1-4	5-10	11-19	20-30	>30
In your lifetime?	☐	☐	☐	☐	☐	☐
In the past year?	☐	☐	☐	☐	☐	☐
In the past month?	☐	☐	☐	☐	☐	☐

b. Please indicate when you first had 5 or more drinks at one time:

☐ Never
☐ Before College
☐ After College

3.
a. How many times, if ever, have you smoked cigarettes?

TIMES	0	1-4	5-10	11-19	20-30	>30
In your lifetime?	☐	☐	☐	☐	☐	☐
In the past year?	☐	☐	☐	☐	☐	☐
In the past month?	☐	☐	☐	☐	☐	☐

b. **Number of cigarettes smoked daily**:

☐ 0/Never ☐ 11-20 ☐ 41-60
☐ 1-10 ☐ 21-40 ☐ >60

4. How many times, if ever, have you smoked marijuana or hashish?

TIMES	0	1-4	5-10	11-19	20-30	>30
In your lifetime?	☐	☐	☐	☐	☐	☐
In the past year?	☐	☐	☐	☐	☐	☐
In the past month?	☐	☐	☐	☐	☐	☐

5. How many times, if ever, have you used cocaine or its derivatives?

TIMES	0	1-4	5-10	11-19	20-30	>30
In your lifetime?	☐	☐	☐	☐	☐	☐
In the past year?	☐	☐	☐	☐	☐	☐
In the past month?	☐	☐	☐	☐	☐	☐

6. How many times, if ever, have you used designer drugs or hallucinogens (eg Ecstasy, GHB, LSD, Mescaline etc)?

TIMES	0	1-4	5-10	11-19	20-30	>30
In your lifetime?	☐	☐	☐	☐	☐	☐
In the past year?	☐	☐	☐	☐	☐	☐
In the past month?	☐	☐	☐	☐	☐	☐

**7. Has your drinking ever caused you to:**	**Never**	**1-2 times**	**3-5 times**	**6-9 times**	**≥10 times**
a. Get behind in your work	☐	☐	☐	☐	☐
b. Call in sick or be late	☐	☐	☐	☐	☐
c. Have trouble getting along with people	☐	☐	☐	☐	☐
d. Worry that you might be using too much or too often	☐	☐	☐	☐	☐
e. Seriously consider suicide	☐	☐	☐	☐	☐
f. Have an auto accident or other type of accident	☐	☐	☐	☐	☐
g. Provide less than your best patient care performance	☐	☐	☐	☐	☐
h. See a psychiatrist, psychologist or a counselor	☐	☐	☐	☐	☐

Section: C

How many times, if ever, have you used following medications/drugs on your own authorization or for use other than intended?

1. Minor opiates (eg: codeine cough syrups)

TIMES	0	1-4	5-10	11-19	20-30	>30
In your lifetime?	☐	☐	☐	☐	☐	☐
In the past year?	☐	☐	☐	☐	☐	☐
In the past month?	☐	☐	☐	☐	☐	☐

2. Major opiates (eg: Fentanyl, Morphine)

TIMES	0	1-4	5-10	11-19	20-30	>30
In your lifetime?	☐	☐	☐	☐	☐	☐
In the past year?	☐	☐	☐	☐	☐	☐
In the past month?	☐	☐	☐	☐	☐	☐

3. Stimulants (eg: Methamphetamine)

TIMES	0	1-4	5-10	11-19	20-30	>30
In your lifetime?	☐	☐	☐	☐	☐	☐
In the past year?	☐	☐	☐	☐	☐	☐
In the past month?	☐	☐	☐	☐	☐	☐

4. Sedative-hypnotics (eg: Temazepam, Flurazepam, Zolpidem)

TIMES	0	1-4	5-10	11-19	20-30	>30
In your lifetime?	☐	☐	☐	☐	☐	☐
In the past year?	☐	☐	☐	☐	☐	☐
In the past month?	☐	☐	☐	☐	☐	☐

5. Tranquilizers (eg: Phenobarbital, Ketamine)

TIMES	0	1-4	5-10	11-19	20-30	>30
In your lifetime?	☐	☐	☐	☐	☐	☐
In the past year?	☐	☐	☐	☐	☐	☐
In the past month?	☐	☐	☐	☐	☐	☐

6. Anxiolytics (Diazepam, Lorazepam, Alprazolam)

TIMES	0	1-4	5-10	11-19	20-30	>30
In your lifetime?	☐	☐	☐	☐	☐	☐
In the past year?	☐	☐	☐	☐	☐	☐
In the past month?	☐	☐	☐	☐	☐	☐

7. Other prescription pain medications (eg: Tramadol, Pentazocine)

TIMES	0	1-4	5-10	11-19	20-30	>30
In your lifetime?	☐	☐	☐	☐	☐	☐
In the past year?	☐	☐	☐	☐	☐	☐
In the past month?	☐	☐	☐	☐	☐	☐

8. If use is >0 any of the questions from 1-7 above then….. please indicate reasons for use (answer all that apply):

- ☐ Supervising self for medical condition
- ☐ For pleasure, curiosity or go along with friends
- ☐ To stay awake, perform better, lose weight etc
- ☐ Don't want to, or can't quit using
- ☐ Originally prescribed but now using on my own

**9. Has your drug use ever caused you to:**	**Never**	**1-2 times**	**3-5 times**	**6-9 times**	**≥10 times**
a. Get behind in your work	☐	☐	☐	☐	☐
b. Call in sick or be late	☐	☐	☐	☐	☐
c. Have trouble getting along with people	☐	☐	☐	☐	☐
d. Worry that you might be using too much or too often	☐	☐	☐	☐	☐
e. Seriously consider suicide	☐	☐	☐	☐	☐
f. Have an auto accident or other type of accident	☐	☐	☐	☐	☐
g. Provide less than your best patient care performance	☐	☐	☐	☐	☐
h. See a psychiatrist, psychologist or a counselor	☐	☐	☐	☐	☐

10. **Do you feel that you have ever used alcohol and other medications/drugs more than you would consider appropriate?**

- ☐ No
- ☐ Yes, Alcohol
- ☐ Yes, Medications/drug use
- ☐ Yes, Alcohol and Medications/drug use

Thank you very much for your participation in the study
